# Supplementary material for: Heme Regulatory Motif of Heme Oxygenase-2 Is Involved in the Interaction with NADPH–Cytochrome P450 Reductase and Regulates Enzymatic Activity
Source: Int J Mol Sci. 2025 Mar 5;26(5):2318. doi: 10.3390/ijms26052318 (PMC11900463; doi:10.3390/ijms26052318)
Supplement: Supplementary file 1 [file ijms-26-02318-s001.zip › ijms-3487634-supplementary.pdf]

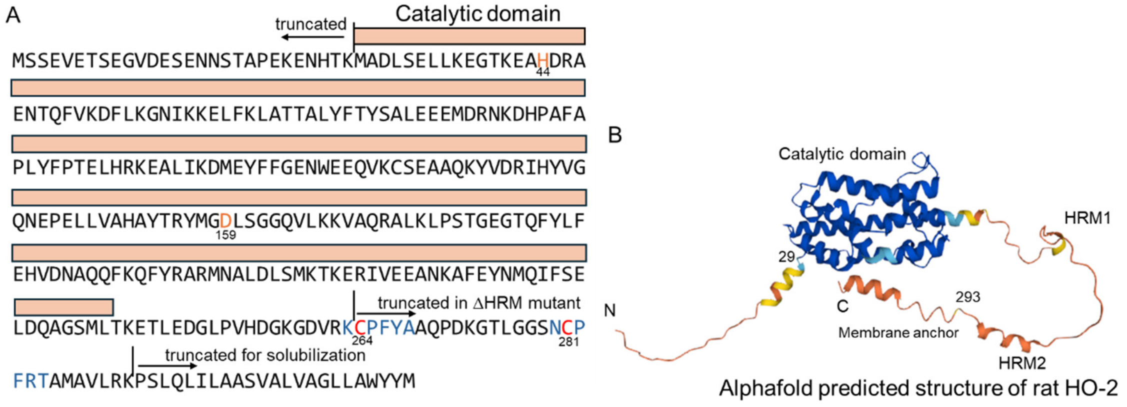

Figure S1. Amino acid sequence and predicted tertiary structure of rat HO-2. A, Amino acid sequence of rat HO-2 (P23711, UniProt) was displayed. Truncated parts in this study were also shown. HRM sequences and Cys264 and Cys281 are colored in blue and red, respectively. The proximal histidine, where the substrate heme is ligated, and the catalytic aspartate residues are colored in orange. B, Alphafold predicted structure shown in the UniProt is displayed. Catalytic domain structure colored in blue is reliable, whereas the other part colored in orange or yellow is unreliable (a per-residue confidence score is lower than 70) and probably unstructured.

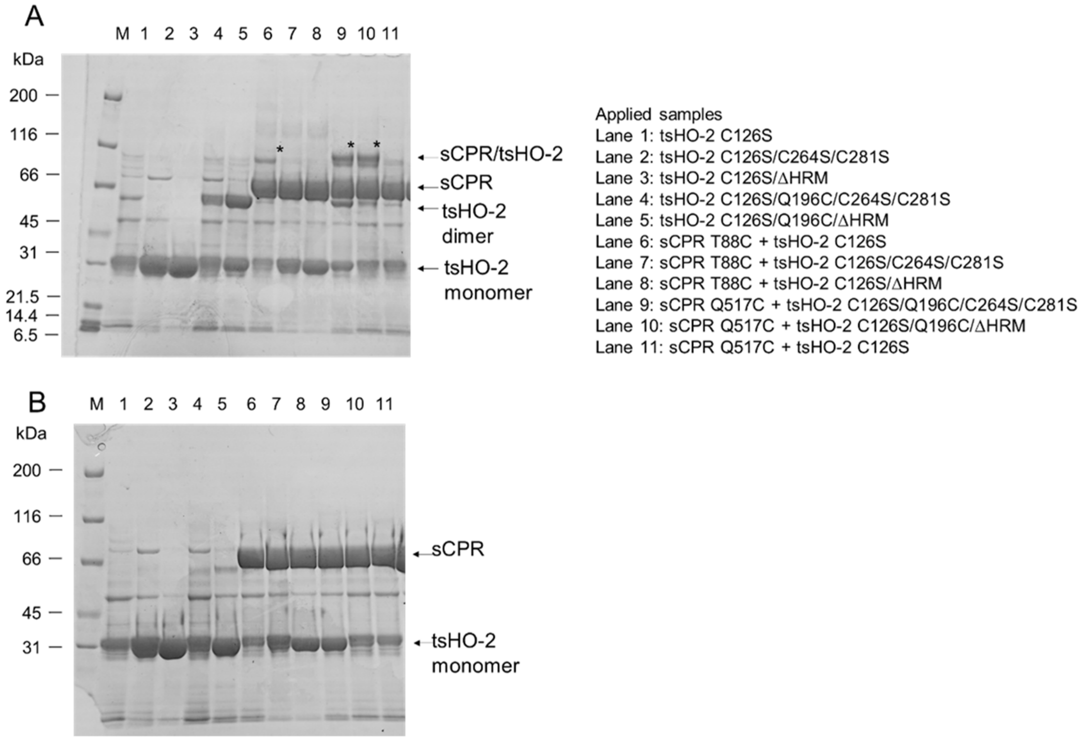

Figure S2. Crosslinking analysis of 6xHis tagged C126S tsHO-2 mutants with sCPR mutants. A, SDS-PAGE in non-reduced condition as shown in Figure 1A. The samples applied to each lane are shown in the right side. Lane M is protein markers. B, SDS-PAGE in reduced condition. The samples applied to each lane were the same as indicated for Figure S2A, but with 2-mercaptoethanol.

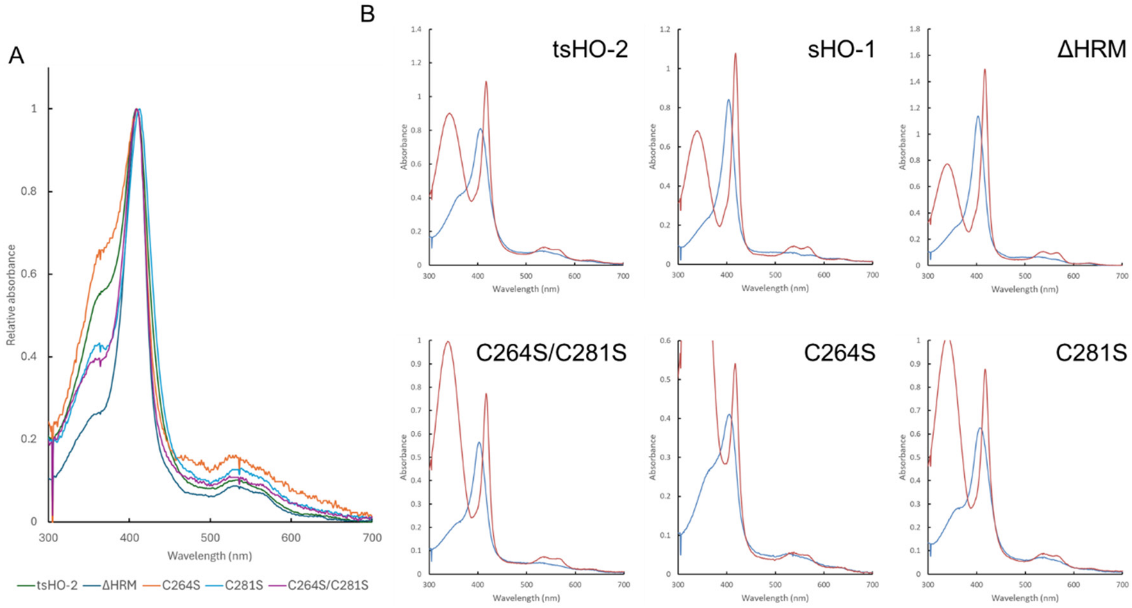

Figure S3. Spectroscopic characters of heme-bound non-His tagged tsHO-2 and its HRM mutants. A, UV-vis spectra of heme-bound tsHO-2 and its HRM mutants in 0.1 M potassium phosphate buffer (pH 7.4). B, UV-vis spectral changes of heme-bound tsHO-2 and its HRM mutants in 0.1 M potassium phosphate buffer (pH 7.4) upon CO binding. Blue and red spectra showed CO-unbound and CO-bound forms, respectively.

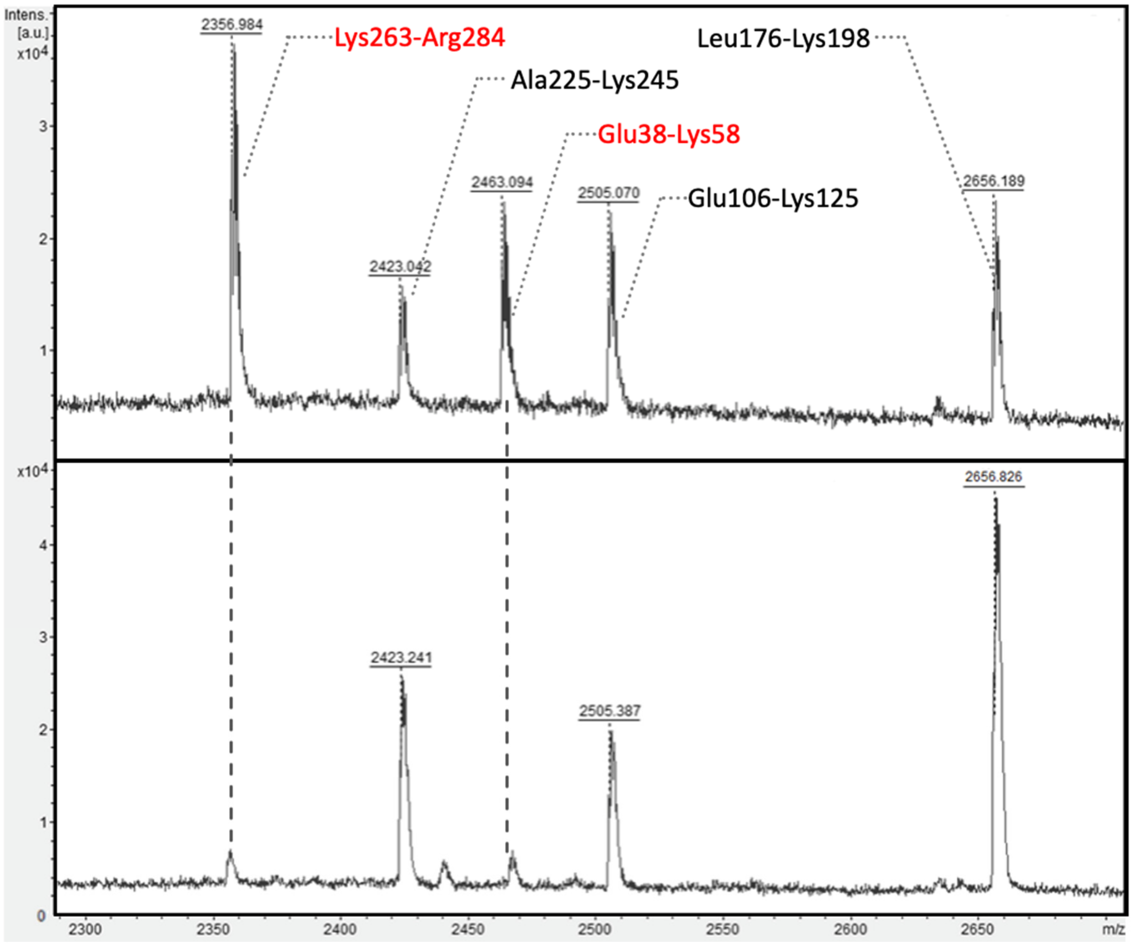

Figure S4. Effect of the heme binding to HRM on enzymatic digestion of tsHO-2. Apo-form (upper panel) and heme-bound form (lower panel) of tsHO-2 were digested with trypsin, then subjected to MALDI-TOF mass spectrometry.

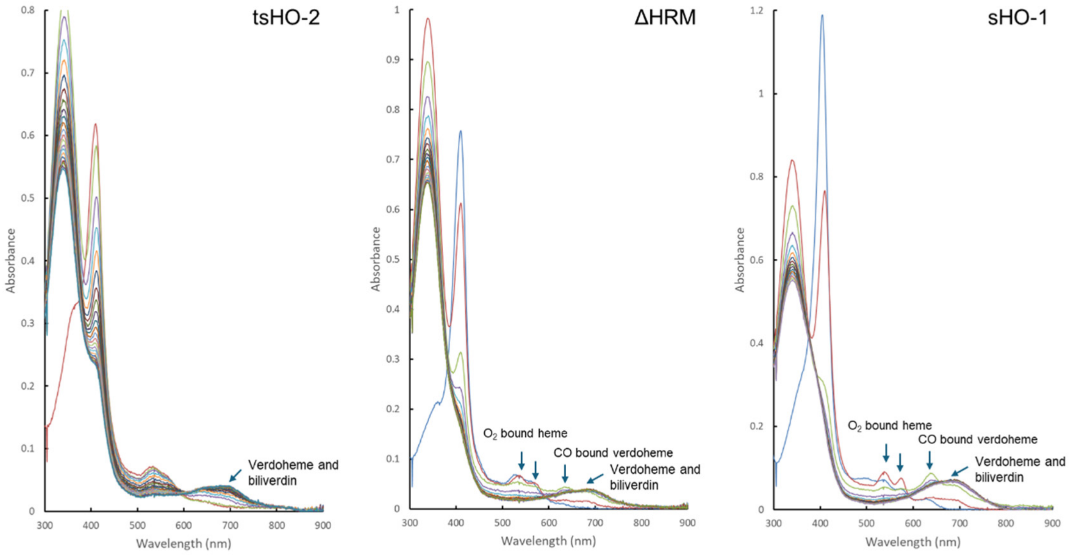

Figure S5. UV-vis spectral changes of heme-bound non-His tagged tsHO-2, ΔHRM, and sHO-1 during single-turnover reaction. Spectra were recorded with 2 min intervals for 40 min in tsHO-2 and ΔHRM, and for 30 min in sHO-1. Apparently the single-turnover reaction of tsHO-2 is slower than those of ΔHRM and sHO-1 although the concentration of CPR (40 nM) was the same. See details in Materials and Methods.

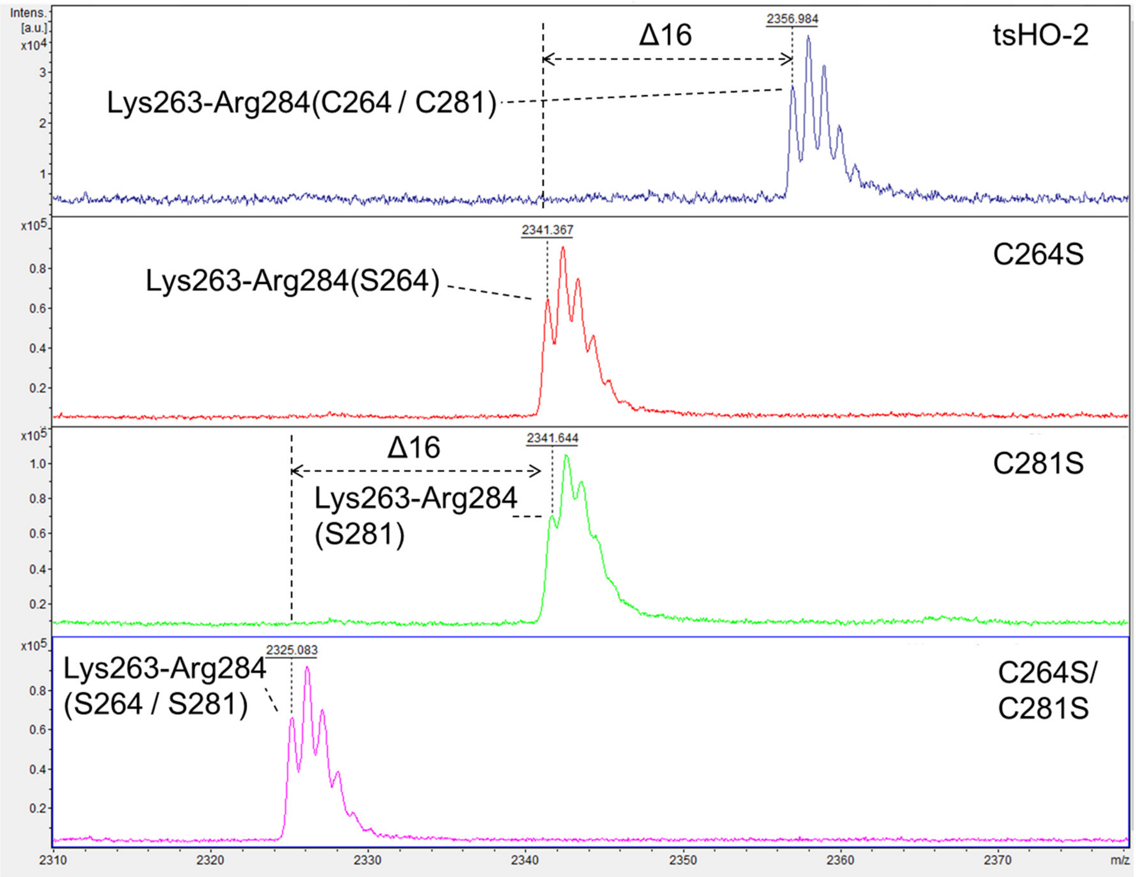

Figure S6. Confirmation of the mutations in the tsHO-2 HRM motifs by MALDI-TOF mass spectrometry. The substitutions of cysteine residues with serine in the HRM of trypsin-digested recombinant protein fragments (residues 263-284) result in a decrease in the mass-to-charge ratio (*m*/*z*) of 16 per substitution. The calculated monoisotopic masses for the fragments from tsHO-2, C264S, C281S, and C264S/C281S were 2357.1, 2341.1, 2341.1, and 2325.1 Da, respectively, and the observed peaks in the mass spectra corresponded to these calculated masses.

Table S1. Kinetic parameters of the decay of the Soret band during single-turnover reactions

	tsHO-2	ΔHRM	C264S	C281S	C264S/C281S	sHO-1
k_{fast} (min^{-1})	0.980 ± 0.134	0.865 ± 0.054		0.296 ± 0.009	0.527 ± 0.012	0.730 ± 0.027
k_{slow} (min^{-1})	0.0864 ± 0.002	0.178 ± 0.016	0.140 ± 0.006	0.0957 ± 0.004	0.104 ± 0.007	
τ_{fast} (min)	1.02 ± 0.15	1.16 ± 0.04		3.38 ± 0.10	1.90 ± 0.04	1.37 ± 0.005
τ_{slow} (min)	11.6 ± 0.03	5.62 ± 0.19	7.13 ± 0.33	10.5 ± 0.1	9.65 ± 0.66	
Ratio of fast phase (%)	21.4 ± 1.2	83.1 ± 1.8	0	59.5 ± 2.5	83.3 ± 1.1	100
R^2	0.9996	0.9997	0.9943	1	0.9999	0.9976

Table S2. Primers used to construct tsHO-2 and its HRM mutants

29-293-f	CATATGGCAGACCTTTCTGAG
29-293-r	GCTGCCGCGCGGCACC
C126S-f	TCCTCCGAGGCTGCCCAGAAGTATGTGGATCGG
C126S-r	CTTCACCTGCTCCTCCCAGTTTTCACCAAAG
C264S-f	AGCCCCTTTTATGCTGCTCAGCCAGACAAAGGTAC
C264S-r	TTTACGTACATCTCCTTTCCCATCATGTACTGGG
C281S-f	AGCCCCTTCCGGACAGCCATGGCTGTGCTGAGG
C281S-r	GTTGCTGCCTCCCAGGGTACCTTTGTCTGGCTGAG
ΔHRM-f	TAACCCTTTTATGCTGCTCAGCCAGACAAAGGTAC
ΔHRM-r	TTTACGTACATCTCCTTTCCCATCATGTACTGGG

Sequences used for mutations are underlined.
